# How our body influences our perception of the world

**DOI:** 10.3389/fpsyg.2015.00819

**Published:** 2015-06-12

**Authors:** Laurence R. Harris, Michael J. Carnevale, Sarah D’Amour, Lindsey E. Fraser, Vanessa Harrar, Adria E. N. Hoover, Charles Mander, Lisa M. Pritchett

**Affiliations:** ^1^Multisensory Integration Laboratory, The Centre for Vision Research, York University, Toronto, ON, Canada; ^2^Department of Psychology, York University, Toronto, ON, Canada; ^3^School of Optometry, University of Montreal, Montreal, QC, Canada

**Keywords:** body representation, distance, gravity, auditory, crossmodal, tactile, self-perception

## Abstract

Incorporating the fact that the senses are embodied is necessary for an organism to interpret sensory information. Before a unified perception of the world can be formed, sensory signals must be processed with reference to body representation. The various attributes of the body such as shape, proportion, posture, and movement can be both derived from the various sensory systems and can affect perception of the world (including the body itself). In this review we examine the relationships between sensory and motor information, body representations, and perceptions of the world and the body. We provide several examples of how the body affects perception (including but not limited to body perception). First we show that body orientation effects visual distance perception and object orientation. Also, visual-auditory crossmodal-correspondences depend on the orientation of the body: audio “high” frequencies correspond to a visual “up” defined by both gravity and body coordinates. Next, we show that perceived locations of touch is affected by the orientation of the head and eyes on the body, suggesting a visual component to coding body locations. Additionally, the reference-frame used for coding touch locations seems to depend on whether gaze is static or moved relative to the body during the tactile task. The perceived attributes of the body such as body size, affect tactile perception even at the level of detection thresholds and two-point discrimination. Next, long-range tactile masking provides clues to the posture of the body in a canonical body schema. Finally, ownership of seen body parts depends on the orientation and perspective of the body part in view. Together, all of these findings demonstrate how sensory and motor information, body representations, and perceptions (of the body and the world) are interdependent.

## Introduction

Since the pioneering philosophical approach of Merleau-Ponty (1945), it has been acknowledged that the senses are embodied. The implication of this approach is that the senses can only be understood by acknowledging the attributes of the body in which they are necessarily situated. In vision, it is obvious that the eyes are in the head and that their viewpoints will be affected by the head’s position and orientation. What is perhaps less obvious is that these properties of the eyes’ vehicle contribute to processing such “visual” judgments as the orientation of the ground plane (Schreiber et al., 2008) and, as we will see, perceived distance. Head position influences the three-dimensional position of the eyes by means of static and dynamic three-dimensional vestibulo-ocular reflexes and through eye height. Information concerning head position is therefore critical to “externalize” the information in the retinal images: that is in creating a representation of the external world. Similar arguments apply to the ears, which are also passengers on the head. Head motion can even help to correctly scale the representation of external space, e.g., the distance between objects, which is notoriously hard to extract from static auditory or visual information alone (Gogel, 1963; Philbeck and Loomis, 1997). Information about the body is also needed to interpret tactile information about the world. When the hands explore and interact with objects in the world, it is necessary to take into account the arrangements of the hands and fingers in order to interpret the patterns of pressure sensed by the fingertips. The representation of the body is also needed to interpret the pressure and location of even simple touches on the skin in order to take into account the uneven density of tactile receptors over the surface of the body in the same way as the visual system must take into account the uneven density of photoreceptors in the retina. In this review we will outline some of the interesting, unexpected and fundamental roles that the body plays in determining our perception of the world.

### The Effect of Body Orientation on Perceived Distance

**FIGURE 1:**
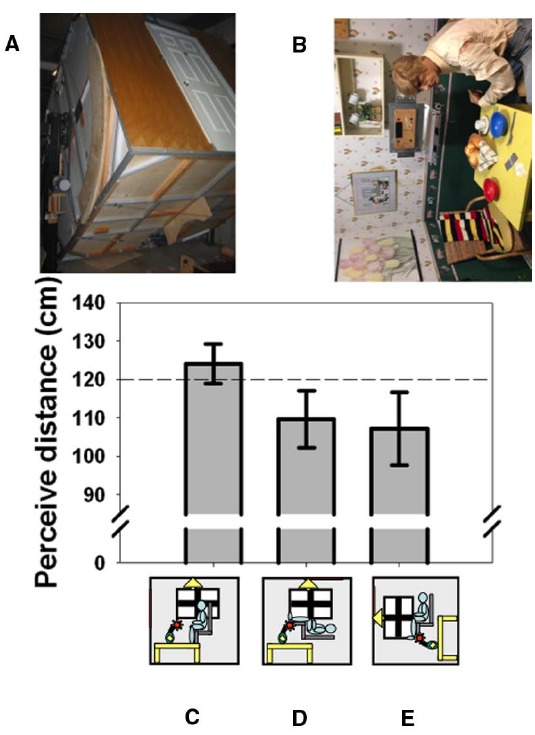
**The effect of body orientation on perceived distance.** When tested in York University’s Tumbling Room Facility **(A,B)**, the perceived distance to the wall opposite was obtained from matching the length of a line projected onto the wall with the length of an iron bar that was only felt **(C)**. The wall was perceived as closer when participants were tilted **(D)** or felt that they were tilted **(E)**. The horizontal dashed line indicates the actual viewing distance. **(A)** shows the room from outside and **(B)** shows the view seen from inside—the mannequin and all the other objects were glued to the inside of the room. Data reanalyzed from Harris and Mander (2014).

Things look different when viewed with the head in an unusual orientation. It is amusing, for example, to look out of a tall building and watch people walking on the street below. Their legs seem to move in a strange way and they often look too small, “like ants,” suggesting a failure in size-distance constancy when looking straight downward which also extends to the perception of speed (Owens et al., 1990). It has long been suspected that body orientation or perceived body orientation may be connected to perhaps the most famous distance-related illusion in psychology: the moon illusion (Rock and Kaufman, 1962). Casual observation shows that the moon appears smaller when it is in the zenith and viewed by looking straight up than when it is close to the horizon and viewed straight ahead. Although the illusion continues to defy complete explanation (Hershenson, 1989; Ross and Plug, 2002; Weidner et al., 2014), it is usually explained with reference to changes in the moon’s perceived distance. We (Harris and Mander, 2014) were the first to measure the effect of posture (and perceived posture) on the perceived distance of objects at biologically significant distances (Cutting and Vishton, 1995), as opposed to the unknowable distance of celestial bodies. We used the York University Tumbling Room facility (Howard and Hu, 2001) in which the orientation of an observer and the surrounding room can be independently varied (Figures 1A,B). We showed that lying supine causes the opposite wall of the room to appear closer than when viewed from an upright position (Figures 1C,D). Rotating the room around an upright observer (Figure 1E) produces an illusion of lying supine (Howard and Hu, 2001). Just *feeling* supine due to this illusion turned out to be sufficient to create this shortening of perceived distance (Harris and Mander, 2014). Thus, it is the perceived orientation of the body that is important in interpreting visual cues to distance. This may be related to the geometrical requirement of taking eye orientation—itself dependent on head orientation—into account in order to interpret binocular cues correctly (Blohm et al., 2008). This unexpected involvement of the body in visual distance perception underscores the importance of the body in interpreting sensory information—it is not a raw sensory signal that leads to perception, but rather the representation in the brain of world features (including the body itself) that is modified in response to sensory input and that determines perception.

### The Effect of Body Orientation on the Perceived Orientation of Objects

**FIGURE 2:**
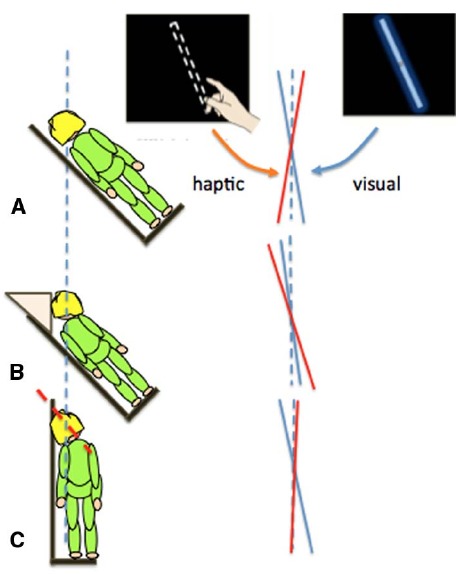
**Different errors in the judgment of gravity vertical in the visual (blue lines) or haptic (red lines) modalities when participants had their whole body (A) or just their torso (B) or head (C) rolled by 45°**.

In the previous section we showed that the body’s orientation in pitch (head over heels) affected distance and size perception. Other changes in self-orientation can also lead to errors in perceptual judgments. When the body is rolled to one side (Figure 2), individuals systematically misperceive the orientation of an object relative to gravity. For example, when judging the orientation of a visual line with gravity vertical, estimates are biased toward the body midline (Aubert, 1886; Mittelstaedt, 1983). In contrast, when setting a bar to gravity vertical using only touch, there is a bias in the opposite direction, away from the body midline (Bauermeister et al., 1964). We (Fraser et al., 2014; Harris et al., 2014) compared visual and manual, touch-based estimates of gravity vertical while the body and head were tilted relative to each other (Figure 2).

Touch-based orientation judgments were affected more by the orientation of the body (Figure 2B), whereas visual errors were largely driven by head tilt (Figure 2C; Guerraz et al., 1998; Tarnutzer et al., 2009). Together, these results show that it is over simplistic to refer to the representation of the body as a single unit when considering the effect of self-orientation on perception. Changes in orientation of the head and body can have different effects on different sensory inputs and so they should be taken into account separately: posture is an important factor.

### The Effect of Body Orientation on Auditory Localization

The ability to localize sound in elevation is tricky. What ability we have depends largely on reflections within the external pinna (Batteau, 1967; Fisher and Freedman, 1968; Makous and Middlebrooks, 1990; Blauert, 1996) and is thus bound to the head. Deducing where sounds are in the external world therefore requires taking into account the position and orientation of the head. Errors in sound localization when the head and body are tilted show that head orientation is only partially taken into account (Goossens and van Opstal, 1999; Parise et al., 2014). In fact, the perceived elevation of a sound, like the perceived orientation of a line we described above, depends on the perceived orientation of the head, which is determined by several factors.

**FIGURE 3:**
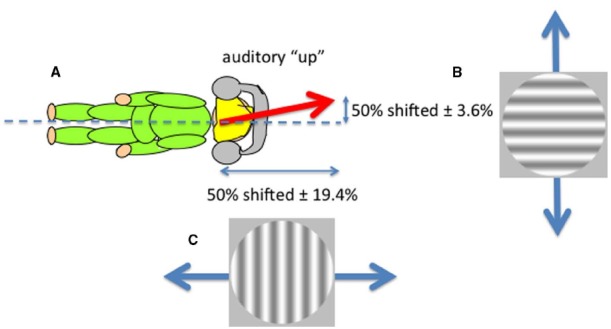
**The perceived direction of auditory “up” (A)** corresponds to the perceptual upright which is determined by a combination of the body idiotropic vector and gravity with more emphasis on the body (Dyde et al., 2006). Non-spatial sounds differing only in their frequency content were used to disambiguate ambiguous visual motion created by superimposing two gratings moving in opposite directions either vertically **(B)** or horizontally **(C)** viewed by an observer lying on their side. The effect of sound in shifting the contrast balance for “ambiguous motion” from 50:50 provided the horizontal and vertical components of the perceived direction of sound.

Sounds that are played through headphones with no intrinsic location at all can nevertheless be perceived as having an elevation by virtue of their frequency content. This is an example of a cross-modal correspondence (Spence, 2011), in this case between pitch and perceived elevation, in which “higher” frequencies are perceived as coming from “higher” in space. But is this elevation defined in head or space coordinates? We showed that such sounds were perceived as lying on an axis defined neither by the head nor gravity but rather that lined up with the perceptual upright (Carnevale and Harris, 2013). Non-spatial sounds (tones played through headphones) that differed only in their frequency content (either rising or falling frequencies) were presented while observers viewed ambiguous visual motion in either the horizontal or vertical directions created by superimposing two gratings moving in opposite directions (left and right or up and down; Figure 3). Observers were tested lying on their sides to separate body and gravitational uprights. A disambiguating effect of sound was found in both directions (up relative to the head and up relative to gravity), suggesting that an auditory upright exists in between the head and gravitational reference frames—a direction very similar to the perceptual upright. The perceptual upright is the orientation in which objects are best identified and represents the brain’s best guess of the direction of up derived from a combination of visual and gravity cues (Dyde et al., 2006) and a tendency to revert to the body midline (Mittelstaedt, 1983). As we showed above for the influence of the body in determining perceived distance and orientation of objects, the perceived orientation of the body also determines the layout of auditory space (see also Parise et al., 2014, who used external sounds). So both visual and auditory perceptions depend on body orientation. What about the perception of touch?

### Tactile Responses Depend on the Direction of Gaze

**FIGURE 4:**
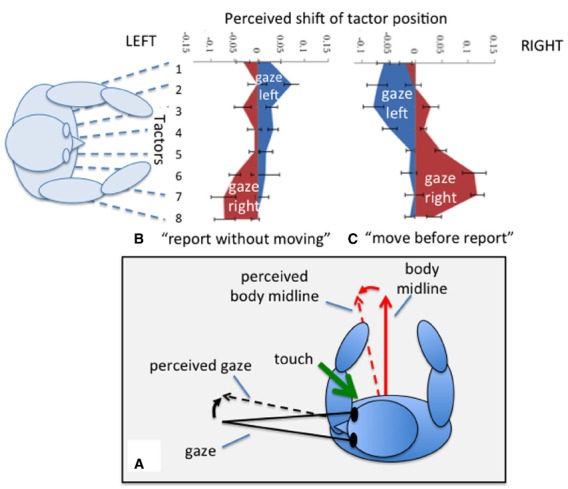
**Localizing a touch on the waist.** During eccentric gaze both the perceived body midline and the perceived direction of gaze are mis-estimated in the directions of the dashed arrows (see text). Localizing a touch relative to one of these reference directions therefore results in the perceived location of touch moving toward that direction **(A)**. For a task in which the location of a perceived touch on the waist is reported without moving gaze, left gaze is associated with a shift (blue area) toward the right and *vice versa* **(B)**. If participants shift gaze before reporting, displacements are in the direction of gaze **(C)**. Data redrawn from Pritchett et al. (2012).

The orientation of the eyes and head are also involved in determining the perceived location of a touch such that the perceived location of a touch is shifted depending on gaze position (Harrar and Harris, 2009; Pritchett and Harris, 2011). Of course the direction of gaze is usually also the point to which attention is directed and attention is known to affect some aspects of tactile perception (Michie et al., 1987) in a way that depends on eye position (Gherri and Forster, 2014). However, Harrar and Harris (2009) found, by overtly orienting attention away from eye position, that attention could account for only about 17% of the effect. Even actions toward a touch are directed toward the shifted perceived position (Harrar and Harris, 2010). The effect appears to be equally affected by either eye or head displacement and is therefore best described as relating to gaze, the sum of eye and head position (Pritchett and Harris, 2011). The perceived location of touch also depends on whether a participant moves their gaze between the presentation of the touch and reporting its perceived location (Pritchett et al., 2012; Mueller and Fiehler, 2014). The perceived location shifts in the same direction as gaze if a gaze change occurs before the report (Harrar and Harris, 2009; Pritchett and Harris, 2011; Harrar et al., 2013), but in the opposite direction if the person does not move before making their report (Ho and Spence, 2007; Pritchett et al., 2012). What do these strange reversals tell us about the involvement of the body in the coding of touch? We can partially explain these gaze-related shifts in terms of the frame of reference in which touch location is coded. The direction of gaze and the direction in which the body is facing are misperceived toward one another when gaze is held eccentrically: the perceived straight ahead of the body is shifted in the direction of gaze, and the perceived direction of gaze is underestimated and perceived as closer to the body’s “straight ahead” (Hill, 1972; Morgan, 1978; Yamaguchi and Kaneko, 2007; Harris and Smith, 2008). Figure 4 shows how the direction in which the perceived location of touch shifts, may depend on whether it is coded relative to one or other of these misperceived reference directions. Displacements in a gaze-centered frame might also be evoked if the location of touch were attracted toward the direction of gaze. We can therefore conclude that touch is initially coded relative to the body midline but, if the location needs to be remembered during a gaze movement, it is switched to a gaze-based reference frame. Touch localization therefore depends on the orientation of the body and gaze. In next section we consider the effect of body size on the perception of touch.

### Tactile Responses Depend on the Perceived Size of the Body

**FIGURE 5:**
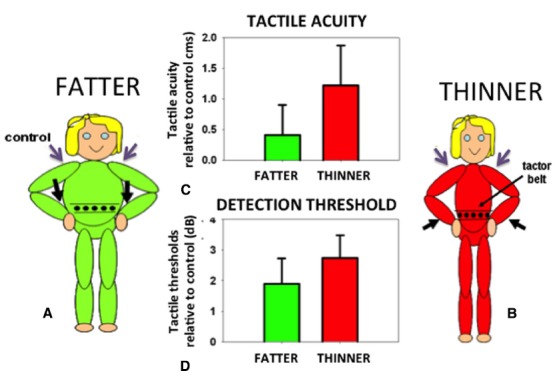
**Participants were made to feel fatter (A) or thinner (B) by vibrating their wrist tendons (black arrows).** Tactile acuity **(C)** and average sensitivity **(D)** on the waist (expressed relative to control trials with vibration on the shoulders, purple arrows) were made worse by either of these manipulations. Data reanalyzed from D’Amour et al. (2015).

In order to identify the size of an object held against the skin it is necessary to correct for the variation in the density of tactile receptors on that part of the body surface. The object will stretch over an array of receptors on the body. The same size object will extend over a different number of receptors depending on the density of receptors in that area of skin. Receptor density must therefore be taken into account if an object’s felt size and proportions are to be accurately determined. In fact, small errors in the perceived size of felt objects are found in which an object felt on an area with a high density of receptors (e.g., the hand) is judged as slightly larger than when the same object is felt on an area with a low density of receptors (e.g., the back). This phenomenon, known as the Weber Illusion (Longo and Haggard, 2011), suggests incomplete compensation for the variation in receptor density and the associated distortions of the homunculus found in the primary somatosensory cortex (Penfield and Boldrey, 1937). The perceived size of the body even in adults is rather plastic and can be altered not only in response to normal growth but also in response to altered feedback concerning body size. For example, the perceived position of a limb can be manipulated by applying vibration to the associated tendon organs. If the affected limb is in contact with another body part, for example the tip of the nose, its perceived location in space will be inferred from the distorted position of the limb. Thus, the nose can appear lengthened: the aptly-named Pinocchio Illusion (Lackner, 1988). Such distortions in the size of a body part are passed on to objects felt on the skin (de Vignemont et al., 2005). If the body part is extended, an object pressed onto the skin is felt as correspondingly longer. Curiously, when perceived body size is distorted in either direction (made either larger or smaller) tactile sensitivity and acuity are both reduced compared to control conditions with non-tendon vibration and attention maintained constant throughout (Figure 5; D’Amour et al., 2015; but cf. Volcic et al., 2013). Distorting the perceived size of the body represents a major change in the critical, universal reference system of the brain: the body. Disrupting the body reference system has multiple fundamental consequences. But what might a reliable body reference look like?

### The Body Reference System

**FIGURE 6:**
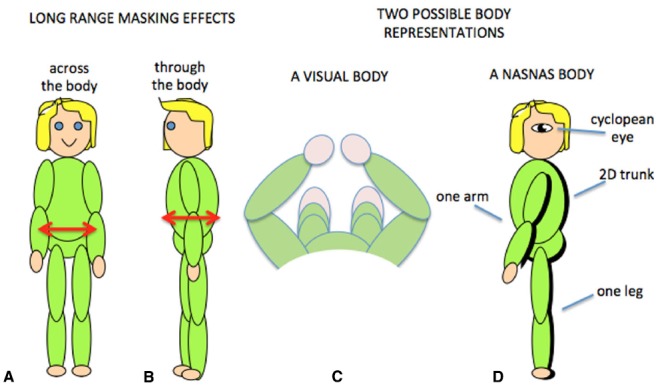
**Long-range tactile masking across (A) and through (B) the body.** Two possible body representations based on the visual view of the body **(C)** or the results of contralateral masking which suggest that at some level the body representation may have a single arm and leg and a 2D trunk **(D)**.

Tactile sensitivity depends on many things. We have demonstrated that it depends on the body representation (Figure 5; D’Amour et al., 2015), and it is very likely that cognitive factors such as attention are also involved (Michie et al., 1987; Spence, 2002; Gherri and Forster, 2012, 2014). An additional factor is that tactile sensitivity can be influenced by simultaneous tactile stimulation on remote areas of the body. This is known as long-range tactile masking (Sherrick, 1964; Braun et al., 2005; Tamè et al., 2011) and seems to indicate a precise connection between the representations of certain patches of skin. For example, the sensitivity to touch on one arm can be influenced by long-range masking only by touch on the corresponding point on the other arm (Figure 6A; D’Amour and Harris, 2014a). Likewise touches on the stomach can be affected by simultaneous touch on the corresponding part of the back (Figure 6B; D’Amour and Harris, 2014b). These effects are quantified relative to when the masking stimulus is positioned at another point on the body so that any attentional effects caused by the presence of a second tactile stimulus were controlled. The question then becomes, how are the “corresponding points” defined and what can they tell us about the nature of the brain’s body schema?

In Head and Holmes’ (1911) original description of the representation of the body in the brain, they postulate a body schema in a “canonical posture” to which the actual posture is later added. The nature of this canonical representation can only be inferred but is presumably based on statistical probabilities of where the various body parts are likely to be (Bremner et al., 2012), that is a prior with the left arm and leg on the left and *vice versa*. This might correspond to the “position of orthopedic rest” (Bromage and Melzack, 1974), the position that astronauts adopt when relaxed in zero gravity^1^ although the detailed layout is hard to access. The prior is likely to rely on visual information about the body (Röder et al., 2004), which might provide a representation of the type shown in Figure 6C although the existence of phantom limbs in people born without arms or legs (Ramachandran and Hirstein, 1998; Brugger et al., 2000) indicates a genetic component to the body schema. Positioning the limbs in a non-canonical position (e.g., crossed) can provide hints about the canonical arrangement. If a touch is applied to the left hand while it is positioned on the right side of the body, saccades toward the touch will often start off directed toward its expected position on the left side (Groh and Sparks, 1996) and reaction times to the touch will be speeded by a visual cue on the left side (Azañón and Soto-Faraco, 2008). More detailed work is required testing many parts of the body (such as the hands, and the upper and lower sections of the limbs) to obtain a more precise impression of the canonical representation. Further, there are likely to be multiple schemas each adapted to a particular aspect of perception (de Vignemont, 2007; Longo et al., 2010).

Obviously the relationship between the front and back of the torso is fixed in all frames of reference, but for the limbs this is not the case. By varying the position of the limbs relative to each other, we have demonstrated that long-range tactile masking also depends on the position of the limbs in space (D’Amour and Harris, 2014a). Such modulation by posture suggests that long-range tactile masking is a phenomenon at or beyond the point at which the postural body schema is derived rather than at or before the level of the primary somatosensory cortex. The connections between the sides of the body has a neurophysiological correlate in which many somatosensory cells with receptive fields on the arms and hands are responsive to stimuli from either side of the body (Iwamura et al., 1994, 1993; Taoka et al., 1998). Such cells thus provide a signal that an arm was touched but do not distinguish which arm: at some level the postural schema seems to have only one arm! There is some indication that cross-body connections might also occur between the legs (Gilson, 1969; Iwamura et al., 2002, 2001) which suggests that this bilateral representation may include the whole body (Figure 6D). We refer to this as a Nasnas body after the monster in *the Book of 1001 Nights*. The Nasnas body may be a somatosensory equivalent to the way that vision is referred to a single cyclopean eye (Mapp and Ono, 1999).

### The Representation of the Body in Defining the Self

**FIGURE 7:**
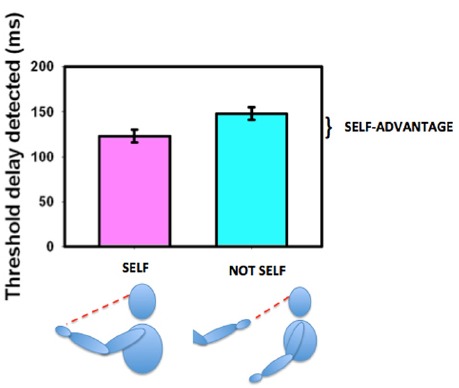
**The self-advantage for detecting a delay between moving one’s own hand and seeing the movement.** When the hand is seen in the natural perspective (“self,” purple bar) the threshold for detecting delay is shorter than when the hand is viewed from the “not self” perspective (cyan bar). The improvement is known as the “self-advantage.” Data redrawn from Hoover and Harris (2012).

The ability to move one’s own body and see that it behaves in the expected way is an important aspect of determining agency (Gallagher, 2000; Tsakiris et al., 2007a,b) and thus in deriving, establishing, and maintaining a sense of ownership of our own body. We established that sensitivity for detecting delay between initiating and seeing a movement was enhanced if the moving body part were seen in its natural orientation (the first-person perspective) as opposed to if it were seen as if it were someone else’s hand (from a third-person perspective, Hoover and Harris, 2012, see Figure 7). This variation with perspective gives us an objective measure of what the brain regards as the body’s first person view. Hand and head movements that are seen from a natural first-person perspective (looking down at the hand or seeing the hand or head in a mirror) are associated with a strong self-advantage, but views of the body from behind or of an arm stretched out toward us in a third-person perspective are not (Hoover and Harris, 2015). This suggests that body parts that can be seen in a first-person perspective are preferentially treated as belonging to us (Petkova et al., 2011b). Parts of the body that cannot be seen directly and thus have no representation from a first-person perspective, such as the back of the body, may not be regarded as parts of the self in the same way as parts of the body that can be seen directly. However, this can altered by providing an unusual first-person visual view of the back (Ehrsson, 2007; Lenggenhager et al., 2007; Spapé et al., 2015) demonstrating the role of learning and experience in forming our perception of our “self.” The suggestion that vision determines what is regarded as self either directly or from the view in a mirror, is compatible with our representation of the body in the brain as having only a two-dimensional representation of the torso as shown in Figure 6D.

## Discussion

This review emphasizes the reciprocal nature of the perception of our bodies in the world and the world that we perceive. Multisensory integration operates not only at the level of integrating redundant cues about object properties—such as when auditory and visual cues signal the location of an event (Alais and Burr, 2004; Burr and Alais, 2006) or when cues about the size of an object are conveyed by both vision and touch (Ernst and Banks, 2002). Multisensory integration also determines the representation of the body in the brain (Maravita et al., 2003; Petkova et al., 2011a), and this representation in turn is fundamental in interpreting all sensory information.

### The Body in the Brain

What is the nature of the body’s representation in the brain? Here we are not considering the consciously accessible representation of the body which may be divided into parts known as body mereology (de Vignemont et al., 2006) with their various cultural associations and accessible to consciousness (Longo, 2014). That is better referred to as a body image. Instead we are attempting to access the internal, possibly monstrous, representation(s) to which all sensory information is related at a neurophysiological level. This representation may be fragmented (Coslett and Lie, 2004; Kammers et al., 2009; Mancini et al., 2011) and apparently illogical in its arrangement. Many converging studies (e.g., Driver and Grossenbacher, 1996; Röder et al., 2004; Soto-Faraco et al., 2004; Longo et al., 2008) suggest that, counterintuitive to the idea of a fragmented, distorted representation, there might be a strong visual component to this representation, at least in normally sighted individuals (see Figure 6C). However, the view of the body is limited in the sense that only some parts can be seen at all and mostly from what we might paradoxically think of as an “odd angle” (see Figure 6C). In which case it is not surprising that there is reduced ownership of the back, which is not directly visible (Hoover and Harris, 2015), and that perception of the back may be closely linked to the more visible front (D’Amour and Harris, 2014b). Representing the three-dimensional body using the two-dimensional flat mapping process that seems to be so common in the brain (Chklovskii and Koulakov, 2004) clearly requires some transformations. It is necessary that sensory inputs are connected appropriately so that for example, a stimulus drawn across the body’s surface is perceived as moving continuously at a constant speed and without discontinuities as it moves from one side to the other or between regions of high and low acuity. That is, it is necessary that the unconscious, distorted body schema be related to the consciously accessible, three-dimensional body image in some.

**FIGURE 8:**
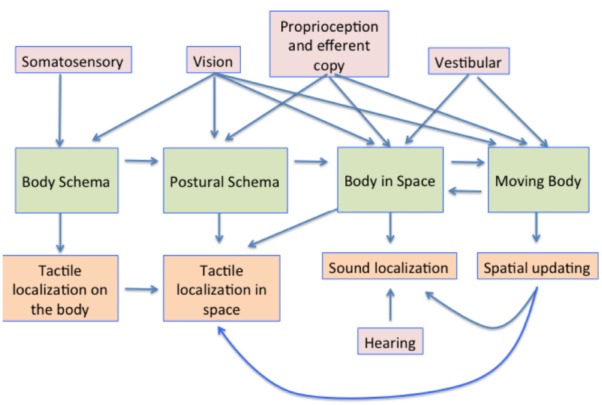
**A summary model of the multisensory contributions to the multiple representations of the body in the brain and how they influence aspects of perception.** The pink boxes show the sensory contributions to the representations shown in the green boxes. These representations are then involved in multiple aspects of sensory processing, some examples of which are shown in the orange boxes below.

The processes involved in creating and using a representation of the body in the brain are summarized in Figure 8. The body schema, in some canonical posture, has posture added to it, using information from proprioception and vision. This representation is then situated in space using proprioceptive vision (vision about the body and its relationship with space) and vestibular cues concerning the direction of up (Harris, 2009). The movement of the body, obtained also from visual and vestibular cues also needs to be taken into account, so that the position of earth-fixed features can be appropriately updated to register their new positions relative to the body both during the movement itself and following repositioning in space.

To consider sensory functioning in isolation of the multisensory context provided by the other senses and without regard to the body of which they are a part has to be regarded as being artificial. It is now the turn of our own bodies to take central stage if we are to understand how we are able to construct our perception of the external world and interact with it.

### Conflict of Interest Statement

The authors declare that the research was conducted in the absence of any commercial or financial relationships that could be construed as a potential conflict of interest.

## Footnotes

1 JSC-09551, Skylab Experience Bulletin No. 17—Neutral Body Posture in Zero G, NASA-JSC, 7–75 cited in http://msis.jsc.nasa.gov/sections/section03.htm
